# Functional Genomics of 5- to 8-Cell Stage Human Embryos by Blastomere Single-Cell cDNA Analysis

**DOI:** 10.1371/journal.pone.0013615

**Published:** 2010-10-26

**Authors:** Amparo Galán, David Montaner, M. Eugenia Póo, Diana Valbuena, Verónica Ruiz, Cristóbal Aguilar, Joaquín Dopazo, Carlos Simón

**Affiliations:** 1 Valencia Node of The National Stem Cell Bank, Centro de Investigación Príncipe Felipe (CIPF), Valencia, Spain; 2 Department of Bioinformatics and Genomics, Centro de Investigación Príncipe Felipe (CIPF), Valencia, Spain; 3 Fundación Instituto Valenciano de Infertilidad (FIVI), Instituto Universitario IVI (IUIVI), University of Valencia, Valencia, Spain; Cincinnati Children's Research Foundation, United States of America

## Abstract

Blastomere fate and embryonic genome activation (EGA) during human embryonic development are unsolved areas of high scientific and clinical interest. Forty-nine blastomeres from 5- to 8-cell human embryos have been investigated following an efficient single-cell cDNA amplification protocol to provide a template for high-density microarray analysis. The previously described markers, characteristic of Inner Cell Mass (ICM) (n = 120), stemness (n = 190) and Trophectoderm (TE) (n = 45), were analyzed, and a housekeeping pattern of 46 genes was established. All the human blastomeres from the 5- to 8-cell stage embryo displayed a common gene expression pattern corresponding to ICM markers (e.g., *DDX3*, *FOXD3*, *LEFTY1*, *MYC*, *NANOG*, *POU5F1*), stemness (e.g., *POU5F1*, *DNMT3B*, *GABRB3*, *SOX2*, *ZFP42*, *TERT*), and TE markers (e.g., *GATA6*, *EOMES*, *CDX2*, *LHCGR*). The EGA profile was also investigated between the 5-6- and 8-cell stage embryos, and compared to the blastocyst stage. Known genes (n = 92) such as depleted maternal transcripts (e.g., *CCNA1*, *CCNB1*, *DPPA2*) and embryo-specific activation (e.g., *POU5F1*, *CDH1*, *DPPA4*), as well as novel genes, were confirmed. In summary, the global single-cell cDNA amplification microarray analysis of the 5- to 8-cell stage human embryos reveals that blastomere fate is not committed to ICM or TE. Finally, new EGA features in human embryogenesis are presented.

## Introduction

Embryonic developmental fate can be described as a progressive loss of totipotency, whose primary outcome is commitment and differentiation into inner cell mass (ICM) and trophoectoderm (TE) [1], [2]. During this period maternal transcripts/proteins control the developmental program until embryonic genome activation (EGA) occurs [3].

Embryo developmental fate in mammals has been investigated mostly in the mouse model, and two distinct hypotheses have been put forward. The first proposes that polarity predetermination takes place before the 2-cell stage where cells adopt differential positions, either inside or outside, depending on the orientation of the cell divisions along the animal-vegetal (AV) axis; thus blastomere fate could be predisposed from fertilization [4]–[11]. The second model stresses that the mouse embryo is entirely symmetric, and that it neither, has an AV nor shows any other pre-patterning. According to this view, 2-cell blastomeres do not differ and their precise contribution to ICM and/or TE cannot be anticipated at this early stage, but later in the morula stage when inner cells contribute to ICM, whereas outside cells differentiate into TE [12]–[17]. In this context, previous studies in the human embryo have also indicated this duality where either the single blastomeres depending on the cleavage planes would determine later developmental fate [18] or the blastomere would exhibit totipotent behaviour during early human development [2].

The genetic mechanisms governing EGA have been well documented in *Caenhorabditis elegans*, *Drosophila melanogaster* and *Xaenopus laevis* [19]–[21]. EGA starts at the 4 to 8-cell stage in human embryos [3], [22], [23] and occurs in a stepwise manner where maternal mRNAs must be depleted, while the transcripts required for growth and differentiation are expressed for the first time, as has been well established in mice [24]–[29]. However, information about this genetic transfer in human embryos is lacking.

Global genome assays provide vast amounts of gene expression profile information and could help to clarify these unsolved scientific issues. Single-cell cDNA microarrays analysis of single blastomeres has been successfully performed in mice [30], [31], but not in humans. Previous studies have revealed not only the ICM gene signature in mouse and human embryos [31], [32], but also stemness [2], [33]–[35], and TE that [36] gene signatures could be used to determine putative differentiation in blastomeres in early preimplantation embryos. Our results indicate that all the blastomeres analyzed in the 5-6- and 8-cell stage embryos show a common transcript pattern, suggesting that cell fate commitment to ICM or TE is still to be determined at these embryonic stages. Furthermore, the embryo genome activation (EGA) process is confirmed at the single blastomere level in the 5-8-cell stage human embryos.

## Results

### Global Gene Expression Profile of Single Human Blastomeres

**Figure 1:**
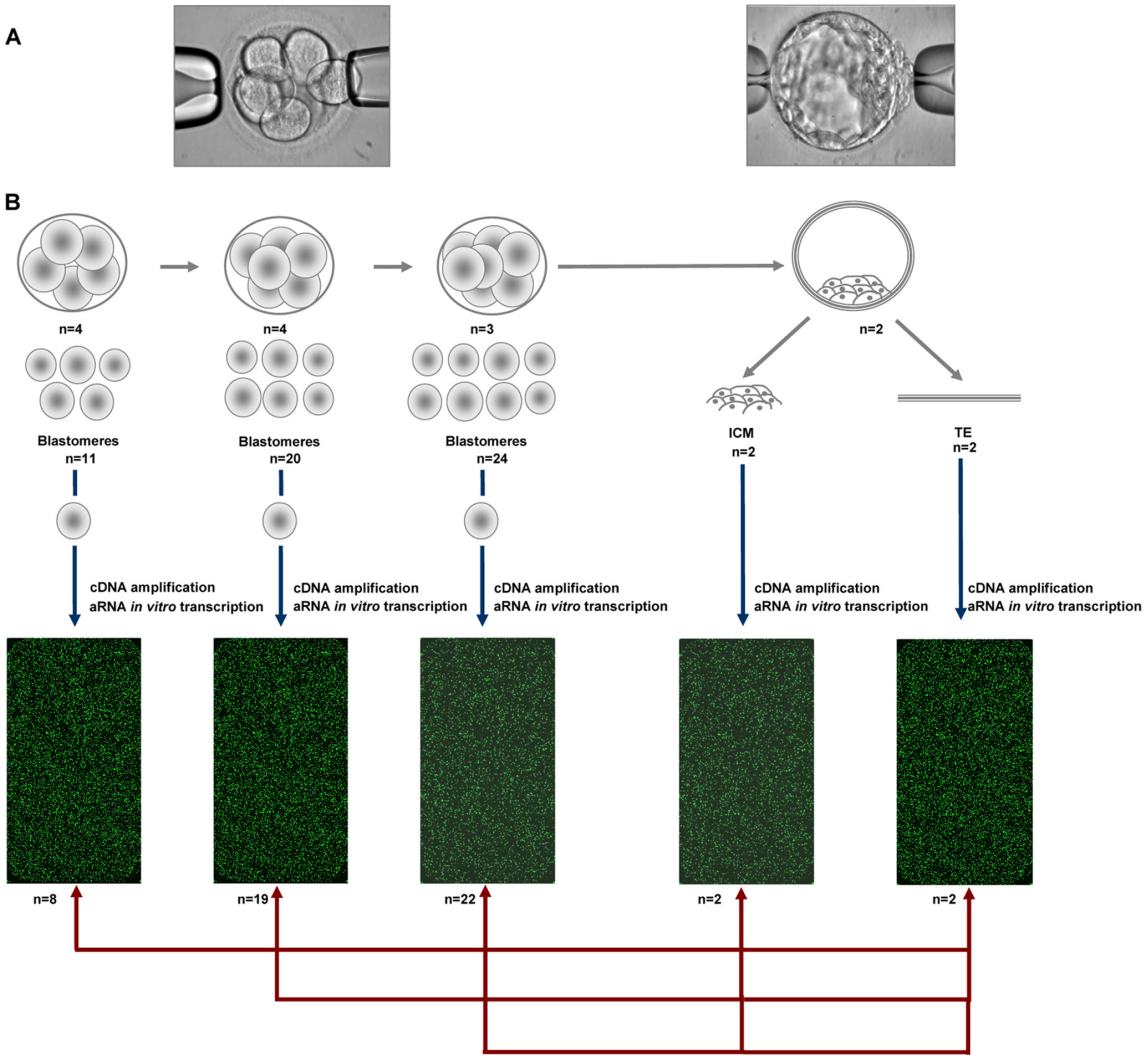
Experimental design. (A) Blastomere biopsy and ICM and TE isolation procedures. (B) Schematic representation of the experimental design. A total of 55 biopsied single blastomeres, 2 ICM and 2 TE were cDNA amplified and *in vitro* transcribed for microarray analysis. The signal quality in the microarrays hybridized to biological samples was compared to that of the negative control, and 6 samples were finally discarded. The normalized data from the 5-, 6- and 8-cell embryos single blastomeres microarrays were compared to each other and with ICM and TE to check for the gene expression related with ICM, stemness, TE and the EGA signature.

A total of fifty-five blastomeres corresponding to 5-cell (n = 4) (11 blastomeres), 6-cell (n = 4) (20 blastomeres), 8-cell embryos (n = 3) (24 blastomeres), and to two blastocysts (n = 2) (2 inner cell masses (ICM) and 2 trophectoderms (TE)) were processed and cDNA amplified as shown in Figure 1. The signal quality in the microarrays hybridized to biological samples was compared to that of the negative control (in water subjected to the same amplification and hybridization protocol). Those arrays whose signal distribution did not significantly differ from the control one according to Wilcoxon's test, were discarded (n = 6). Finally, 53 samples were kept in the study and used in the gene expression analysis, including 8 blastomeres from 5-cell, 19 from 6-cell, 22 from 8-cell, 2 ICM and 2 TE from blastocyst stage embryos (Figure 1). For convenience, we use the term “gene” instead of “gene feature” in the microarray data descriptions.

A total of 26,504 data points were assigned from known and novel genes, including transcripts that are specific to ICM, stemness and TE. Unsupervised hierarchical clustering mainly distinguished two groups, one consisting in blastocysts and the other from single blastomeres (not shown). Differential gene expression analysis was performed comparing 5-, 6-, and 8-cell embryos between them as well as with ICM and TE samples. Limma t-statistic methodology, followed by a Benjamini & Hochberg p-value correction was computed using Babelomics web tool (see Methods). A significance level of 0.05 in the corrected p-values was used to call genes differentially expressed. Numerous significant differences were found when comparing 5-, 6-, and 8-cell embryos with the ICM- and TE-isolated and amplified samples but fewer when comparing the 5- to 8-embryos among them as revealed by the gene set analysis (Table 1; Table S1).

**Table 1:** General gene set analysis.

		1	0	−1
KEGG	E8.E5	0	43	0
	E6.E5	0	43	0
	E8.E6	0	43	0
	ICM.E8	20	14	9
	ICM.E6	17	17	9
	ICM.E5	13	27	3
	TE.E8	19	13	11
	TE.E6	23	7	13
	TE.E5	16	20	7
Biological Process	E8.E5	0	255	11
	E6.E5	1	238	27
	E8.E6	0	266	0
	ICM.E8	155	78	33
	ICM.E6	166	66	34
	ICM.E5	113	134	19
	TE.E8	142	86	38
	TE.E6	143	85	38
	TE.E5	103	140	23
Molecular Function	E8.E5	0	186	3
	E6.E5	7	172	10
	E8.E6	0	189	0
	ICM.E8	96	46	47
	ICM.E6	93	52	44
	ICM.E5	58	111	20
	TE.E8	98	50	41
	TE.E6	101	47	41
	TE.E5	59	114	16
Cellular Component	E8.E5	1	101	16
	E6.E5	2	89	27
	E8.E6	0	118	0
	ICM.E8	73	28	17
	ICM.E6	71	29	18
	ICM.E5	50	57	11
	TE.E8	71	28	19
	TE.E6	72	28	18
	TE.E5	44	66	8

Number of significant KEGG, biological process, molecular function and cellular component terms in each comparison. 1 means over-representation of the term in the first class, 0 means no significant terms, and -1 means over-representation of the term in the second class. Significant terms appear neither in the KEGG analysis nor in any 6-8-cell stage embryos comparison. The complete gene set analysis can be found in Supplemental Table S1.

The same pattern of strong differences between blastomeres and ICM and TE samples and of weak differences among blastomeres was found in the Gene Set Analysis for the Gene Ontology terms and the KEGG pathways using the Babelomics functional profiling tools (see Methods). No KEGG pathways and very few GO terms were enriched when comparing 5-, 6-, and 8-cell embryo blastomeres among them. Of those, the most relevant functions involved RNA and DNA processing and were enriched in 5-cell compared to 6-, and 8-cell embryo blastomeres. This might be indicative of the maternal mRNA degradation and the beginning of embryonic gene activity. When comparing 5-, 6-, and 8-cell embryos with ICM and TE, most diverse biological functions were found significantly enriched, indicating strong functional differences between these two embryonic developmental stages.

Global gene expression values were also employed to investigate whether a single blastomere could have a differential transcriptome in the 5- to 8- cell embryos. For that purpose, the correlation among all the blastomeres from the same complete embryo (intra-embryo) or from different embryos (inter-embryo) was compared. Mann Whitney, Students t- and Kolmogorow-Smirnov tests all agreed in that general intra-embryo correlation (>0.8) was significantly higher than inter-embryo (>0.7) correlation. This suggests that differences among the transcriptomes from blastomeres in the same complete embryo are much less than differences between embryos, and herein no potential blastomere commitment at the 5- to 8- cell stage can be inferred.

### Blastomeres from 5 to 8-cell stage embryos show a common ICM, stemness and trophectoderm gene signature

#### Housekeeping profile pattern

**Figure 2:**
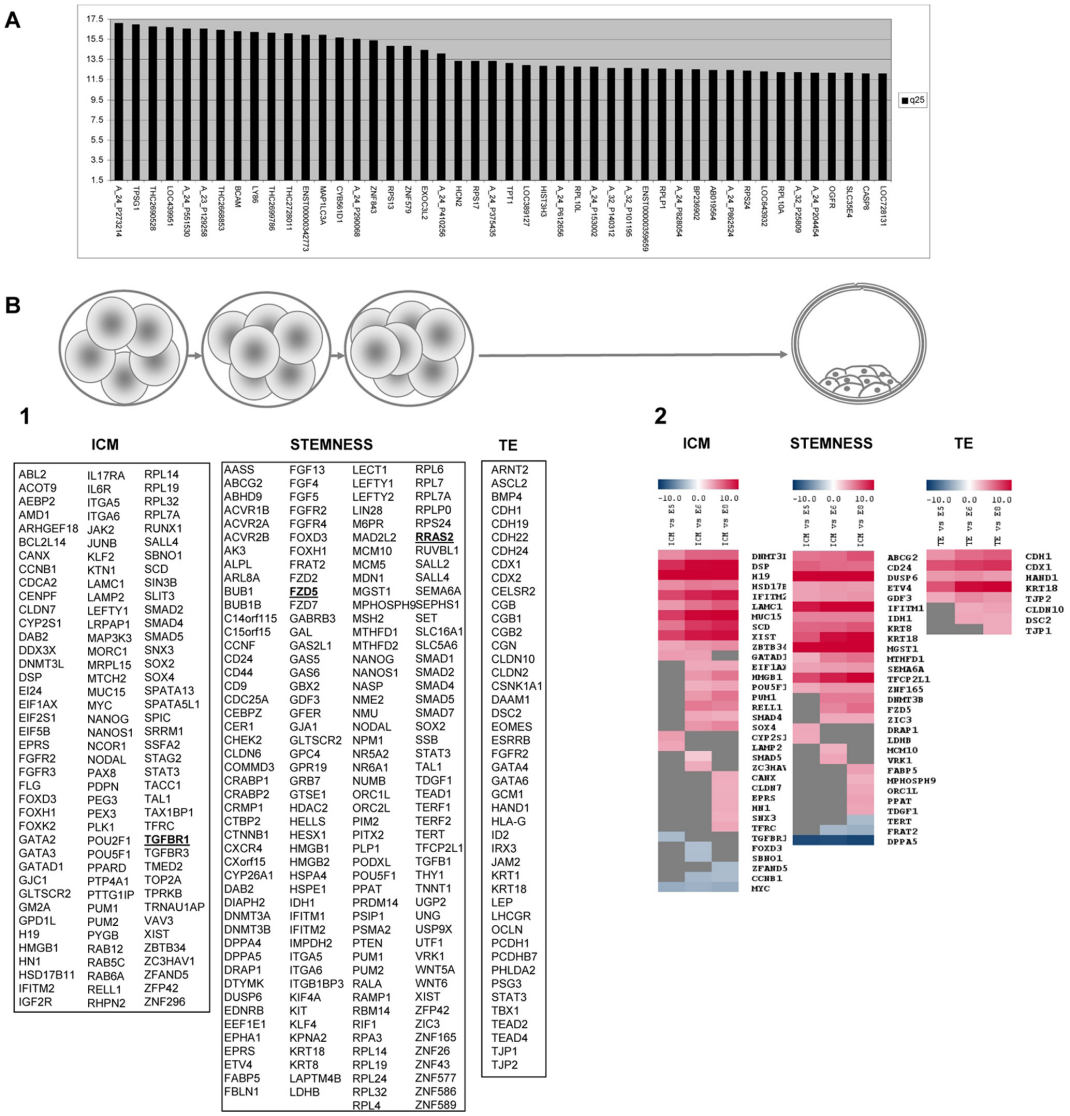
Gene expression analysis and blastomere fate. (A) List of the putative housekeeping genes common for blastomeres, and the ICM and TE cells from the blastocysts. Those genes with higher values and a low standard deviation (sd iqr <1) (values corresponding to quantile 25 with a sd iqr less than 1) were chosen as putative housekeeping genes (X axis). The maximum data score value in the microarray is 17.5 and 1.63 the minimum (Y axis). (B) Comparative analysis of ICM, stemness and TE gene markers in the single blastomeres from the 5-, 6- and 8- cell embryos (1) were compared to the ICM and TE differentiated cells (2). (1) No statistical difference (p value<0.05) was found for most markers except for *RRAS2*, *FZD5* and *TGFBR1* (underlined and in bold), which were up-regulated in 5-cell embryo blastomeres compared to the 6-cell (*RRAS2*), 8-cell (*FZD5*), and to both of them (*TGFBR1*). (2) Heat map representation of the significant differential expression (p value<0.05) between the 5- and 8- single blastomeres with ICM and TE. Gray indicates no significant differences. Red means an overexpression in the ICM or TE samples, and blue depicts an over-representation in the single blastomere samples. The color code of the expression level is indicated at the top of the figure. The complete data can be checked in Supplementary Table S2.

Housekeeping genes should be generally highly expressed and stable, meaning not variable, across samples. To search for genes accomplishing the first condition, we kept only those that had a gene expression measurement above the 3^rd^ quartile of the intensity distribution in all arrays. To find those that, in addition were stable, we kept the genes having low standard deviation as well as low interquartile range (sd, iqr <1). (Figure 2A). Forty-six genes corresponding to the structural constituent of ribosome (*RPL10L*, *RPLP1*, *RPS13*, *RPS24*, *RPL10A*, *RPS17*, *A_24_P375435*, *ENST00000359659*), the cell surface (*A_24_P551530*, *BCAM*), ion channels (*CYB561D1*, *HCN2*, *TPT1*), the endopeptidase activity (*TPSG1*, *CASP8*), related to inflammatory response (*LY86*), autophagy (*MAP1LC3A*), DNA topological change (*EXOC3L2*) and chromatin (*HIST3H3*), cyclin-dependent protein kinase activity (*A_32_P101195*), and regulation of cell growth (*OGFR*), were identified (Figure 2A). The inclusion of traditional reference genes such as *HPRT1*, *GAPDH* and *ACTB* [37], [38], [39] was discarded. Furthermore, 13 human novel genes recently described as universal markers, including *ARL18B*, *CTBP1*, or *ZNF207* [38] were not included in the selected list. Finally, one gene belonging to the group of genes related with ribosomal proteins, RPS24, was selected as the housekeeping gene for the validation assays in this study.

#### ICM gene profile

One hundred and twenty genes previously reported to be characteristic of human ICM [1], [2], [32], [40] and mouse ICM [24], [31] signatures were analyzed in the single blastomeres from the 5- to 8- cell stage embryos (Table S2). A cross-species analysis for human homologs was done. ICM markers including *DDX3*, *DNMT3L*, *FOXD3*, *JAK2*, *LEFTY1*, *MYC*, *NANOG*, *POU5F1*, *RPL19*, *SOX2*, *XIST* or *ZFP42* displayed no significant differences between either the blastomeres belonging to the same stage embryo or the different cell stages, except *TGFBR1* which was overexpressed in the 5-cell stage embryos compared to the 6- and 8-cell stage embryos (Figure 2B1). As expected, when the single blastomeres from the 5-, 6- and 8- cell stage embryos were compared to ICM, significant differences were found in 34 genes (Figure 2B2). Twenty-eight genes were overexpressed in ICM, including *DNMT3L*, *POU5F1*, *SMAD4* or *XIST*, and 6 were overexpressed in blastomeres, namely *TGFBR1*, *FOXD3*, *SBNO1*, *ZFAND5*, *CCNB1*, and *MYC*.

#### Stemness gene profile

The stemness gene signatures composed of 190 genes [1], [2], [25], [32]–[35], [41]–[48] were used to compare the blastomeres from the 5-, 6- and 8- cell stage embryos (Table S2). All the genes were present in the array, and no significant differences were found between either the blastomeres from the same stage or different stages, except *RRAS2* and *FZD5* which were overrepresented in the 5-cell embryo blastomeres compared to those from the 6- and 8- cell embryos respectively (Figure 2B1). Characteristic stemness genes including *NANOG*, *DNMT3B*, *GABRB3*, *GDF3*, *SOX2*, *POU5F1*, *ZFP42*, and *TERT* were all detected in every blastomere analyzed irrespectively of the developmental stage. Furthermore, a group of markers were selected as they were quite stably and highly expressed in all 49 blastomeres, namely *DPPA5*, *FOXD3*, *HDAC2*, *RPL19*, *RPL4*, *TERT*, *THY1* and *UTF* (Table S2). Therefore, these could be used as a molecular set to identify the blastomeres from the 5- to 8-stage developmental embryos. When the blastomeres from the three developmental stages were compared to ICM amplified samples, 26 were found to be significantly overexpressed in ICM versus blastomeres such as *GDF3*, *KRT18*, and *TDGF1*, among others, and 3 were highly expressed in blastomeres, including *TERT*, *FRAT2*, and *DPPA5*. The common studied genes for both ICM and stemness were *IFITM2*, *XIST*, *HMGB1*, *POU5F1*, *PUM1*, *SMAD4*, *SMAD5*, *EPRS*, and *FOXD3*. It is interesting to note that *KRT18* has been described for both stemness and TE signatures (Figure 2B2).

#### Trophectoderm gene profile

To check for primary differentiation to the TE, 45 genes which have been previously reported to be involved in its differentiation were investigated in all the single blastomeres from the 5- to 8-cell stage embryos [1], [2], [18], [25], [33], [36], [40], [45], [49]–[51] (Table S2). The cross-species homologs from mice were selected whenever necessary. No significant differences were observed among all the blastomeres from the same and different (5-, 6- or 8-cell) stage embryos, and all the markers, including *GATA6*, *EOMES*, *CDX2*, *CDH24* or *LHCGR*, displayed a score in the microarrays (Figure 2B1) (Table S2). When the data values from single blastomeres were compared to those obtained from the isolated TE from blastocysts, 8 gene markers were found to be highly expressed in the TE samples including *CDH1*, *CDX1*, and *KRT18*, among others (Figure 2B2).

By taking these considerations together, the gene expression signature of ICM, stemness and trophectoderm showed no strong differences between blastomeres from the 5- to 8- cell stage embryos, while clear differences with differentiated ICM and TE were displayed, thus suggesting no fate commitment at this early developmental embryonic stage.

### Transcriptomics of the embryonic genome activation (EGA)

**Figure 3:**
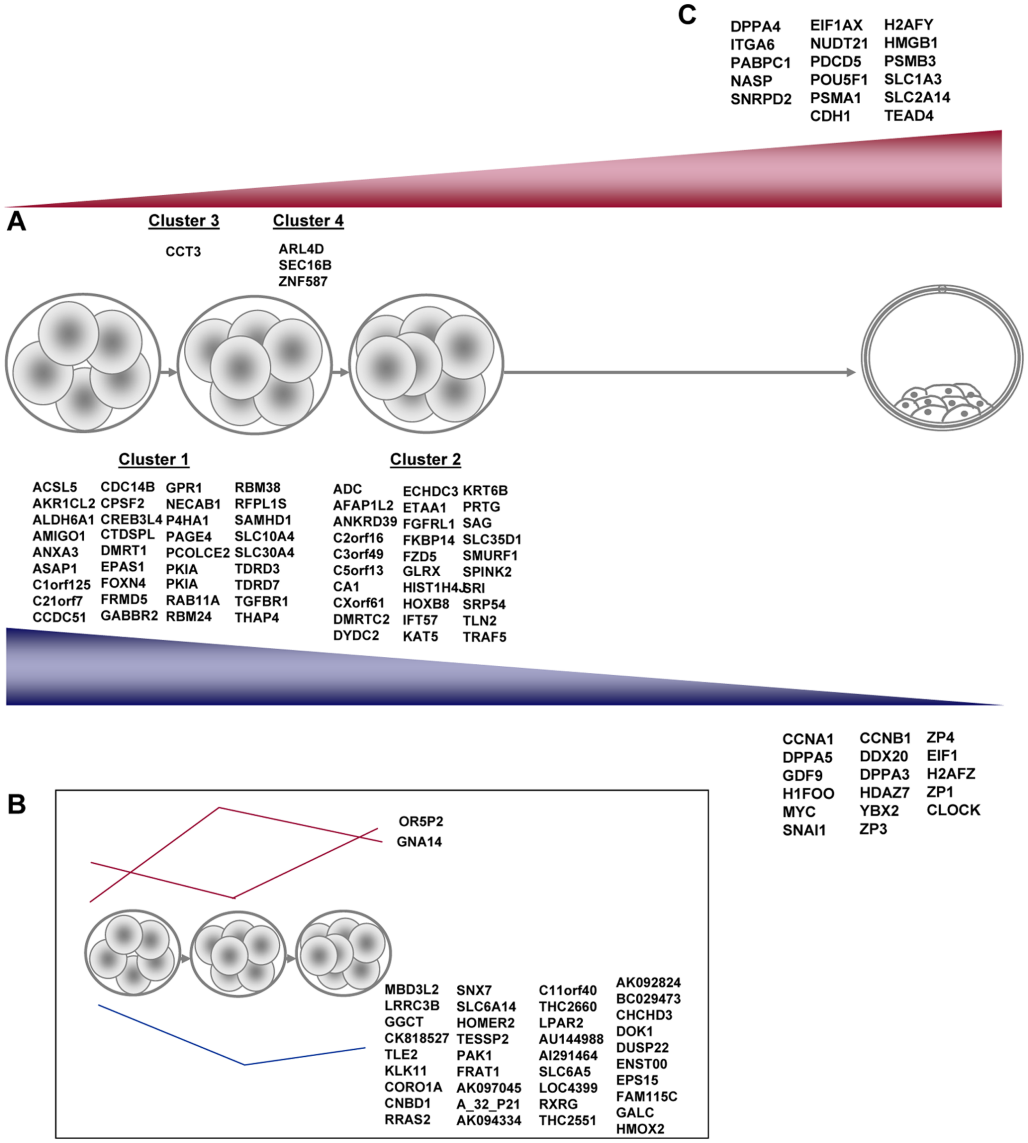
Genes involved in embryonic genome activation (EGA). EGA entails maternal degradation from the genes existing in the blastomeres (shown in blue) and the gene activation of the transcription factors of the embryo genome (shown in red). (A) The novel gene markers involved in putative maternal degradation or genome activation in the 5- to 8- cell embryo blastomeres have been grouped into clusters according to their gene expression profile. (B) List of markers showing an intermediate pattern in the 5- to 8- cell embryo blastomeres, including maternal degradation at the 6-cell and slight activation later, gene activation at the 6-cell stage and slight subsequent down-regulation and a significant overexpression in the 8-cell stage versus the 6-cell-stage blastomeres. (C) Definitive analysis of EGA involving the 5- to 8- cell embryos compared to blastocysts. Gene expression profiles confirm previously reported results. Only those genes with statistically significant differences (p value<0.05) have been considered for the analysis. The complete data can be checked in Supplemental Table S3 and Supplemental Figure S1.

Embryonic genome activation (EGA) is a two-fold process that requires maternal mRNA degradation and embryonic gene activation between the 4- to 8-cell embryos in humans. In our study, a comparative gene expression profile between the 5- to 8-cell embryos at the single blastomere level revealed a novel signature of EGA genes including 147 genes related with maternal transcriptional depletion, and 6 genes involved in embryo genome activation (Figure 3AB; Table S3). This is in agreement with previous studies which demonstrate that maternal gene down-regulation far outweighs embryonic gene up-regulation [22]. Based on their expression profiles, genes were joined in clusters (Figure 3A). Those genes which underwent maternal degradation in the 6-cell stage embryo (e.g., *AMIGO1*, *ANXA3*, *TGFBR1*) were considered to be Cluster 1, while Cluster 2 included the significantly down-regulated genes at the 8-cell stage (e.g., *DMRTC2*, *FZD5*, *SMURF1*). Gene activation involved *CCT3* (Cluster 3) starting at the 6-cell stage embryos, and Cluster 4 including the genes activating at the 8-cell stage (*ARL4D*, *SEC16B*, *ZNF587*). It is interesting to note that three other groups displayed an intermediate pattern with those genes showing maternal degradation, gene activation with a subsequent partial down-regulation and gene expression depletion with final activation (Figure 3B; Figure S1).

Furthermore, 92 human and mouse genes, which have been previously described to take part in EGA, were chosen to be compared in blastocysts versus the single blastomeres from the 5- to 8-cell embryos (Table S3). Definitive maternal transcript degradation was represented by 17 genes including *DPPA5*, *MYC* or *CLOCK*, among others (Figure 3C), and the final embryonic genome activation was confirmed by the overexpression of 17 genes including *EIF1AX*, *POU5F1* or *TEAD4* among others (Figure 3C), which have already been described in other animal species [11], [22]–[27], [52]–[60].

### Microarray data validation

**Figure 4:**
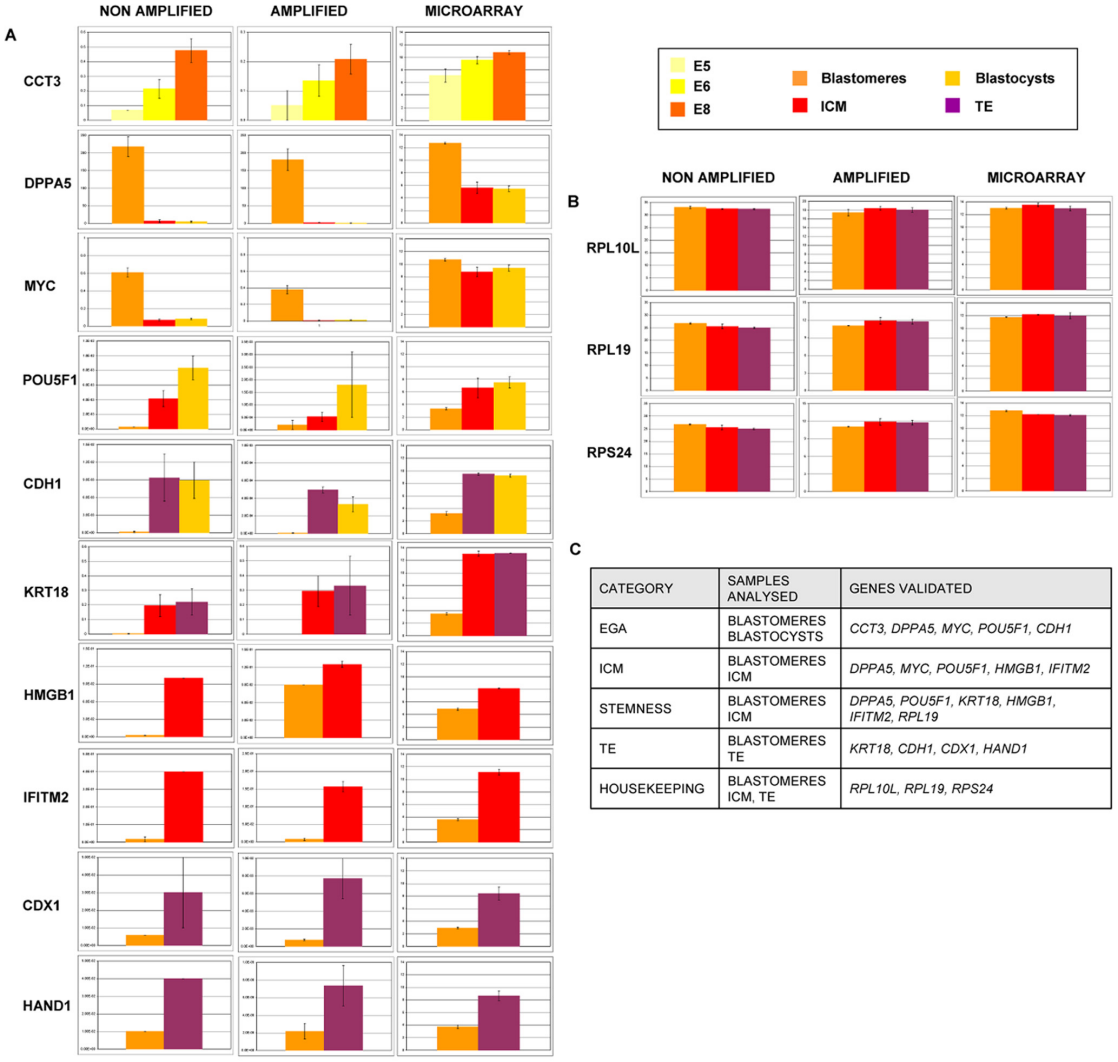
Gene expression validation of the microarray data by qPCR. (A) Gene expression analysis was analysed in non amplified and amplified samples in 5-, 6- and 8-cell embryo blastomeres with *CCT3* primers, blastomeres and ICM (*HMGB1*, *IFITM2*), blastomeres and TE (*CDX1*, *HAND1*), blastomeres, ICM and TE (*KRT18*), blastomeres, ICM and blastocysts (*DPPA5*, *MYC*, *POU5F1*), and blastomeres, TE and blastocysts (*CDH1*). *RPS24* was used as the reference gene in all cases. Results were compared with microarray data to check ICM, stemness, TE and EGA signatures. (B) Cp values of the selected housekeeping genes in non amplified and amplified blastomeres, ICM and TE samples and their microarray counterparts. (C) Gene list description for each category validation. Color legends used in the graphics is specified at the top right of the Figure.

Gene expression validation was performed by quantitative PCR (qPCR) using the amplified cDNA from the microarray surplus and novel samples, as well as the non amplified cDNA from single blastomeres, ICM and TE. Specifically, qPCR assays were performed for a total of 13 genes including ICM markers (*POU5F1*, *DPPA5*, *MYC*, *HMGB1*, *IFITM2*), stemness (*POU5F1*, *DPPA5*, *KRT18*, *IFITM2*, *HMGB1*, *RPL19*), TE (*KRT18*, *CDH1*, *HAND1*, *CDX1*), EGA (*DPPA5*, *POU5F1*, *CCT3*, *MYC*, *CDH1*) and the putative housekeeping markers common to blastomeres, ICM and TE (*RPS24*, *RPL10L*, *RPL19*). *RPS24* was used as the housekeeping reference gene in all cases. Most of the amplified blastomere cDNA from the 5-, 6- and 8- cell embryos (n = 11, 20, and 24, respectively), the 2 ICM and 2 TE used in the microarray were used for the qPCR experiments. Furthermore, 2 more biopsied ICM and TE from blastocysts were also amplified. Non amplified samples also included 5-cell (n = 4, 4 blastomeres), 6-cell (n = 2, 11 blastomeres), and 8-cell (n = 4, 30 blastomeres) embryos, as well as the blastocysts (n = 5) biopsied for ICM and TE isolation. In summary, the expression profile analysis of the genes investigated confirmed microarray results (Figure 4).

## Discussion

Understanding human preimplantation development from a global genome perspective is crucial for basic embryology research, regenerative medicine because of the derivation of human embryonic stem cells (hESC) from single blastomeres [61], [62], and for translational applications in reproductive medicine such as preimplantation genetic diagnosis (PGD) [1], [63], [64].

To investigate cell fate in blastomeres from the 5- to 8-cell stage embryos, we first looked at the global gene profile in all the blastomeres from the same embryo, which resulted in a higher intra-embryo correlation as compared to the inter-embryo association. Next, we searched for a differential gene signature among the blastomeres from the 5-, 6- and 8-cell stages according to the previously described gene patterns of ICM and TE [1], [31], [32], [36], [40], and stemness [62], [65], [66]. Markers of stemness are responsible for controlling undifferentiation and immortality in hESCs, thus they could be candidate genes for making developmental decisions in early human embryos [2], [34]. One hundred and twenty ICM genes, 190 stemness and 45 TE markers were used to provide a wide spectrum to characterize the blastomeres at the single cell level. All the blastomeres from the 5-, 6- and 8-cell embryos displayed expression levels for ICM, stemness and TE markers, and no statistical differences were found among them, except for *TGFBR1*, *FZD5* and *RRAS2* significantly overexpressed in the 5-cell embryo blastomeres. Thus, gene expression appeared to be similar in the single blastomeres analyzed independently of both embryo-cell stage and origin. Furthermore, a set of stably and highly expressed genes was established to create a blastomere signature composed of the *DPPA5*, *FOXD3*, *HDAC2*, *RPL19*, *RPL4*, *TERT*, *THY1* and *UTF* genes.

Additionally, single blastomere gene profiles were analyzed with those obtained from isolated ICM and TE. First, a common housekeeping gene expression for the three sample types was established. This signature included a set of 46 genes involved in ribosomal, cell surface and enzyme proteins, among others, and conferred a unique reference pattern to human embryos (e.g., *RPS24*, *RPL10L* or *RPL13*). Previously characterized housekeeping genes like *GAPDH* and *ACTB*, or the novel universal reference genes (e.g., *ARL18B* or *ZNF207*) [39] were not included in our housekeeping human embryo signature. When performing a comparative analysis of the gene signatures between the isolated blastomeres, ICM and TE, significant differences were found for the characteristic markers *POU5F1* or *DNMT3B*, which were highly expressed in ICM, or *DPPA5* and *FOXD3* which were up-regulated in the blastomeres, indicating a unique gene expression pattern for blastomeres and blastocysts. According to this differential pattern, hESCs derived from different sources may have specific properties. The immortality gene marker *TERT* (Telomerase) and the stemness keeper *MYC* were over-represented in the single blastomeres if compared to ICM. Since *TERT* is involved in the gradual decrease in the potential for unlimited proliferation, and as *MYC* has an activating effect on telomerase [67], [68], the hESCs derived from the 5- to 8-cell stage blastomeres are expected to have increased potential for propagation *in vitro*.

On the other hand, the highly expressed genes in ICM involved characteristic stemness transcription factors such as *POU5F1*, *PUM1*, *GDF3*, *ETV4*, *FZD5, ZNF165* or *TGF1*, interferon-induced transmembrane proteins *IFITM1* and *IFITM2*, as well as members of the intermediate filament gene family *KRT8* and *KRT18*, which are also characteristic of TE differentiation. Of special interest are those genes involved in methylation, a crucial epigenetic modification for embryonic development, imprinting and X-chromosome inactivation. De novo methylation was observed in the blastocyst to be restricted to ICM [69]. The differential analysis in our study has revealed that de novo methylases *DNMT3A/B/L* not only show a higher expression in ICM if compared to blastomeres, but also their transcriptional regulated gene *H19* and the X inactivation *XIST* gene, thus confirming the expected putative transcriptome and suggesting potential differences in hESCs in accordance with their origin.

In parallel, one of the most important embryo developmental outcomes at the 5- to 8-cell stage is embryonic genome activation (EGA) [22]. EGA failure leads to embryo arrest and eventual implantation failure. Three main requirements need to be met for successful genome activation, namely maternal mRNA degradation, embryonic gene transcription activation and epigenetic changes. The differential expression profile between the 5- 6- and 8-cell embryos demonstrates a putative novel EGA gene signature, and shows a vast number of transcripts which are down-regulated at the 6- and 8-cell levels if compared to the 5-cell stage embryo (n = 147), plus a low number of up-regulated genes during this period (n = 6). Maternal down-regulated genes include those relating with the Wnt cannonical pathway (*FZDZ5*, also related with stemness, *TLE2*, *FRAT1*) and the FGF/TGFB signaling pathways (*TGFBR1*, *FGFRL1*, *SMURF1*), cell proliferation and development (*HOXB8*, *RRAS2*, also related with stemness, *DUSP22*), methylation (*MBD3L2*), apoptosis (*PAK1*, *DUSP22*), the G-protein family (*GABBR2*, *FZD5*, *LPAR2*), cytoskeleton structure (*AFAP1L2*, *KRT6B*, *TLN2*, *RRAS2*, also related with stemness, *HOMER2*, and *PAK1*), immune response (*ANXA3*, *AFAP1L2*) and histones (*HIST1H14J*) among others. The sex determination genes *DMRT1* and *DMRT2* were also found to be depleted. No activated gene presented a common ontological pattern except *OR5P2* and *GNA14* which belong to the G-protein family.

Further assays comparing the 5-8-cell embryo blastomeres to the blastocysts for the previously described genes involved in EGA confirmed this developmental process. The maternal depletion enclosed genes that are involved in development and pluripotency (*DPPA3*, *DPPA5*, also involved in stemness, *SNAI1*, *DDX20*, *MYC*), related with transcriptional processes (*DDX20*, *EIF1*, *CLOCK*), are associated with germ cell specification and meiosis to spermatogenesis (*CCNAI*, *DDX20*, *ZP1*, *ZP3*, *ZP4*), to oocytes (*GDF9*, *H1FOO*, *CCNB1*) and to both (*YBX2*), and also related with histones (*H2AFZ*). Furthermore, we also found that the previously described gene transcription activation markers include cell proliferation, pluripotency and development-related genes (*DPPA4*, *ITGA6*, *NASP*, *POU5F1*, *TEAD4*) cell differentiation markers (*CDH1*, *TEAD4*, *SLC1A3L*, *SLC2A14*), transcriptional activation processes (*PAPBPC1*, *SNRPD2*, *EIF1AX*, also related with stemness, *NUDT21*, *HMGB1*, *TEAD4*), apoptosis (*PDCD5*, *CDH1*, *PSMB3*), histones (*H2AFY*, *NASP*), and immune response (*PSMA1*). This list of genes that were down- and up-regulated during the activation of the zygote genome is a valuable tool which could be used in future studies into the basic molecular mechanisms determining the normal development of the pre-implantation embryo.

In conclusion, this is the first global genome study performed on single human blastomeres at an early developmental stage. In whole genomic comparison terms, and regarding a comparative study conducted with more than 400 genes characteristic of ICM, stemness and early differentiation to the trophectoderm, our results indicate that blastomere fate in mRNA expression terms is not determined at the 5–8 cell stage. EGA outcome has also been assessed by genome-wide and comparisons made between established gene pattern strategies at the single blastomere level.

## Materials and Methods

### Ethical statement

This study was approved by the institutional review board of the Prince Felipe Research Centre. Permission for this Project was granted by the Spanish Authority, Instituto de Salud Carlos III (ISCIII) on December 13, 2006. Human embryos frozen at different stages at the Instituto Valenciano de Infertilidad (IVI) were donated for this work according to Spanish law 45/2003. Progenitors were asked to sign a specific consent form for stem cell derivation as indicated in Royal Decree 2132/2004.

### Thaw and embryo culture

Day 3-stage human embryos were thawed using the Thaw Kit (Vitrolife, Sweden) and incubated in pre-equilibrated CCM medium (Vitrolife, Sweden) in a highly humidified incubator with 5% CO_2_ in air for at least 2 hours before the biopsy. All the embryos used for the blastomere biopsy were grade I or II according to a standard scoring system (embryos with blastomeres of equal size with little or no cytoplasmic fragmentation) [70].

### Blastomere Biopsy

Multiple blastomere biopsies were obtained from each embryo following the single-cell biopsy procedure which is similar to that used in the preimplantation genetic diagnosis (PGD). For micromanipulation purposes, GPGD medium supplemented with 5% HSA was used (Vitrolife, Sweden). Briefly, blastomeres were suctioned by making an approximately 50 µm diameter hole in the zona pellucida with a specific 50 µm biopsy pipette (HumaGene Inc, Scottsdale, USA), while the embryo was held with a holding pipette. After pulling out the pipette entirely from the embryo, the blastomere was expelled gently in the biopsy medium drop. This procedure was repeated for each blastomere contained in all the embryos. Separated blastomeres were washed 4 times in pre-equilibrated CCM medium and placed separately in a lysis buffer for the RNA extraction assay.

### Inner Cell Mass (ICM) and Trophoectoderm (TE) isolation

Thawed day 3-stage embryos were cultured until day 6 in pre-equilibrated CCM medium in a highly humidified incubator at 37°C and 5% CO_2_. Blastocysts were scored according to Gardner and Schoolcraft [71]. All the blastocysts used to obtain inner cell mass (ICM) and trophectoderm (TE) separately were cavitated or expanded with ICM grade A–B and TE grade A–B. ICM and TE isolation was carried out using a micromanipulator with holding micropipettes (Humagen, Charlottesville, VA) and an inverted microscope. The blastocyst was placed in a drop of GPGD medium (Vitrolife) supplemented with 5% Human Serum Albumin (Vitrolife) in a micromanipulation plate (Beckton & Dickinson) to be later held with the holding pipettes from both sides in an attempt to localize ICM at the 9 o'clock position. ICM was separated from TE by 20–30 infrared laser pulses (200 mW × 0.5 ms, Zilos-tk™, Hamilton Thorne Biosciences) by applying laser shots perpendicularly to the pipettes near ICM, and by paying special attention not to damage it. When both parts were separated, the zona pellucida was separated by careful pipetting, and ICM and TE were placed separately in lysis buffer for subsequent RNA extraction.

### RNA amplification

Global mRNA amplification was performed following a previously reported protocol with slight modifications [30]. The biopsied single blastomeres collected in cell lysis buffer and containing oligo (dT)-tagged primer V1 (dT)_24_, were incubated for 90 sec at 70°C, for cell lysis and RNA denaturalization. First-strand cDNAs were synthesized by retro-transcription (RT) with the SSIII (Invitrogen) for 20 min at 50°C and later inactivation at 70°C for 10 min. After RT, the remnant primer was degraded by exonuclease I (Takara Bio, Japan) at 37°C for 30 min and a later inactivation at 80°C for 25 min. cDNAs were then tailed with poly(dA) by terminal deoxynucleotidyl transferase (TdT) (Invitrogen, CA, USA) at 37°C for 15 min and a later inactivation at 70°C for 10 min. The cDNA products were divided into four tubes, and the second strand of the poly(dA)-tailed cDNAs was synthesized with another oligo(dT)-tagged primer,V3(dT)_24_ in an initial 1-cycle PCR reaction consisting of 95°C for 3 min, 50°C for 2 min, and 72°C for 3 min, followed by a second 20-cycle PCR reaction consisting of 95°C for 3 sec, 67°C for 1 min, and 72°C for 3 min plus 6 additional seconds per cycle, and a final extension for 10 min at 72°C. PCR products were put together and purified with the DNA Clean & Concentrator™ Kit (Zymo Research, CA, USA), eluted with 30 µl of double distilled water (Gibco BRL, CA, USA) and quantified with a Nanodrop spectrophotometer (NanoDrop Technologies, DW, USA). In this step, amplified cDNA products were checked by qPCR with a ribosomal gene (*RPL19*) to positively identify amplified cells. Then, purified products were divided into 8 tubes, and 3 µl of each purified cDNA were subjected to another PCR amplification reaction containing the primers V3dT_24_ and T7V1 bearing the T7 promoter, consisting of 1 cycle of 95°C for 5 min and 30 sec, 64°C for 1 min and 72 °C for 18 sec, 10 cycles of 95°C for 30 sec, 67°C for 1 min, and 72°C for 5 min and 18 sec plus 6 additional sec per cycle, and a final extension for 10 min at 72 °C. PCR products were then purified with the DNA Clean & Concentrator™ Kit (Zymo Research, CA, USA) and eluted with 30 µl of double distilled water (Gibco BRL, Carlsbad, CA). Purified products were electrophoresed and gel purified with the ZymoClean™ Gel DNA Recovery Kit (Zymo Research, CA, USA) and eluted in 10 µl of Double Distilled Water (Gibco BRL, CA, USA). Purified products were divided into 4 tubes, and 3 µl of each purified cDNA were subjected to a final PCR cycle with primers bearing the T7 promoter sequence (T7V1 and V3dT_24_ primers) consisting of 95°C for 5 min and 30 secs, 67°C for 1 min and 72°C for 16 min. PCR products were put together and purified with the DNA Clean & Concentrator™ Kit (Zymo Research, CA, USA). Purified cDNAs quantity and quality were determined using the NanoDrop ND-1000 spectrophotometer (Nanodrop Technologies, DW, USA) and the 2100 Bioanalyzer (Agilent Technologies, CA, USA), respectively.

### Microarrays Labelling and Hybridization

Supplied cDNAs were used to produce Cyanine 3-CTP-labeled cRNA using the Quick Amp Labelling Kit, One-Color (Agilent Technologies, CA, USA). The ‘One-Color Microarray-Based Gene Expression Analysis’ protocol Version 5.7 (Agilent Technologies, CA, USA) was followed. The protocol was implemented as follows: to 10 µl of cDNA, 4 µl of 5× First Stand Buffer and 6 µl of H_2_O were added. cDNA was labelled (2 hours or 16 hours) in the IVT reaction with T7 RNA polymerase (Ambion, Austin, USA) and the resulting cRNA was purified. Yield and Cy3 specific activities were measured by spectrometry (Nanodrop Technologies, DW, USA) while cRNA length was evaluated by an mRNA 6000 Nano Bioanalyzer (Agilent Technologies, CA, USA) assay. Hybridization was carried out following the ‘One-Color Microarray-Based Gene Expression Analysis’ protocol Version 5.7 (Agilent Technologies, CA, USA). Hybridization products were incubated for 17 hours or 20 hours at specified temperature. Those slides considered to have good and consistent hybridizations were subjected to bioinformatic analyses. The data files have been deposited in the NCBI Gene Expression Omnibus (GEO, http://www.ncbi.nlm.nih.gov/geo/) and are accessible through GEO series accession number GSE 22032. Results validation was achieved by quantitative PCR.

### Bioinformatic analyses

Samples were first filtered by comparison to the negative sample microarray. Global intensity measurements of 6 microarrays were no different to that of the water hybridized, thus these 6 samples were considered to have no signal and were therefore excluded from the analysis. The other 53 microarrays were left for the analysis. The Agilent Processed Signals (Agilent Feature Extraction Software: http://www.chem.agilent.com/enus/products/instruments/dnamicroarrays/featureextractionsoftware/pages/default.aspx) were standardized across arrays using quantile normalization [72]. A differential gene expression analysis was carried out using the limma [73] package from Bioconductor (http://www.bioconductor.org/). Multiple testing adjustment of the p-values was done according to the methodology of Benjamini and Hochberg [74]. The gene set analysis was carried out for the Gene Ontology terms and for the KEGG Pathways using a logistic regression model [75], [76] GO annotations for the genes in the microarrays were taken from the Ensembl 55 (http://www.ensembl.org) release and KEGG Pathways from the KEGG web page (http://www.genome.jp/kegg/). All analysis methods and databases where used as implemented in Babelomics web tools [77] (http://www.babelomics.org/).

### Real-time PCR for microarrays validation

To verify the microarray data, real-time polymerase chain reaction (qPCR) was used to check the gene expression profiles of several target genes on the same samples used in the microarray and in the cDNA from single biopsied and amplified blastomeres, biopsied ICM and TE, amplified and non amplified. All the qPCR reactions were performed in a Lightcycler® 2.0 (Roche Applied Science, Germany) according to standard procedures. Next, 2 µl of each sample were subjected to an initial denaturalization program (95°C) for 10 min, followed by 45 amplification cycles consisting in 10 sec at 95°C, an annealing step at 59°C for 6 sec and a 10 sec extension step at 72°C where the SYBR GreenI (Roche Applied Science, Germany) was visualized. Samples were later subjected to a melting program to check for specific amplification. All the qPCR experiments were normalized to the housekeeping gene, RPS24. Each lot of experiments was performed at least in triplicate and included a calibrator to check experimental variation. The primers used for the validation are listed in Supplementary Table S4.

## Supporting Information

Table S1 Gene set analysis summary results for KEGG, Biological process, Molecular function and cell components for 5-, 6- and 8-cell embryos comparing single blastomeres among them and comparing each, 5- to 8-cell embryo blastomeres with ICM and TE.
(0.12 MB XLS)

Table S2 Adjusted p values resulting for E5 (5-cell embryo single blastomeres) E6 (6-cell embryo single blastomeres) and E8 (8-cell embryo single blastomeres) comparison and among E5 E6 and E8 with ICM, for ICM and stemness signature, and with TE values for TE signature. Adjusted p values <0.05 were considered to be significant. Normalized data for single blastomeres, and ICM or TE, are also supplied.
(0.43 MB XLS)

Table S3 Significant adjusted p values (<0.05) showing novel EGA genes as a result of E5 E6 and E8 comparison, and for previously reported markers when E5, E6 and E8 were compared to blastocysts (p values<0.05).
(0.04 MB XLS)

Table S4 List of primers used in this study for microarray data validation by qPCR.
(0.04 MB DOC)

Figure S1 EGA signature. Heat map representation of the significant differential expression between the 5–8 single blastomeres showing novel EGA genes (A) and compared with blastocysts for previously reported genes (B). Red means an overexpression in the blastocyst samples, and blue depicts an overexpression in the single blastomere samples. The color code of the expression level is indicated at the top of the figure.
(0.65 MB TIF)
