# Supplementary material for: Functional Genomics of 5- to 8-Cell Stage Human Embryos by Blastomere Single-Cell cDNA Analysis
Source: PLoS One. 2010 Oct 26;5(10):e13615. doi: 10.1371/journal.pone.0013615 (PMC2964308; doi:10.1371/journal.pone.0013615)
Supplement: Table S4 — List of primers used in this study for microarray data validation by qPCR. (0.04 MB DOC) [file pone.0013615.s004.doc]

| **Marker** | **Sequence** | **Amplicon** | **Characteristic** |
| --- | --- | --- | --- |
| **DPPA5** | CGGATCTCGAATCCCTTACA  ACTGGAGCATCCACTTGGTC | 137 | ICM/Stemness/EGA |
| **POU5F1** | AGCAAAACCCGGAGGAGT  CCACATCGGCCTGTGTATATC | 114 | ICM/Stemness/EGA |
| **HMGB1** | TGCGAAGCTGAAGGAAAAAT  CTTCCTCCTCCTCCTCATCC | 161 | ICM/Stemness |
| **MYC** | CAGATCAGCAACAACCGAAA  GGCCTTTTCATTGTTTTCCA | 168 | ICM/Stemness/EGA |
| **IFITM2** | ACGGAACTACTGGGGAAAGG  CTGCTCCTCCTTGAGCATCT | 146 | ICM/Stemness |
| **CDX1** | TCGGACCAAGGACAAGTACC  ATCTTCACCTGCCGTTCAGT | 147 | TE |
| **CDH1** | CAGCACGTACACAGCCCTAA ACCTGAGGCTTTGGATTCCT | 159 | TE/EGA |
| **HAND1** | ACATCGCCTACCTGATGGAC AGGGCAGGAGGAAAACCTT | 148 | TE |
| **KRT18** | CACAGTCTGCTGAGGTTGGA  GAGCTGCTCCATCTGTAGGG | 164 | TE/Stemness |
| **CCT3** | GGAGACGGCAGTTCTGCTAC  CCTTGCCTAGCACTCACTCC | 122 | EGA |
| **RPS24** | TCCTCCTTGGCTGTCTGAAG  CGGGGTGAAGGACATCAAT | 124 | Housekeeping |
| **RPL19** | CGAATGCCAGAGAAGGTCAC  CCATGAGAATCCGCTTGTTT | 157 | Housekeeping |
| **RPL10L** | ACCAAGCTTCAGAACGAGGA  CTTCTTGGCCACCATGTCTT | 147 | Housekeeping |
