# Supplementary figures and images for: Functional Genomics of 5- to 8-Cell Stage Human Embryos by Blastomere Single-Cell cDNA Analysis
Source: PLoS One. 2010 Oct 26;5(10):e13615. doi: 10.1371/journal.pone.0013615 (PMC2964308; doi:10.1371/journal.pone.0013615)

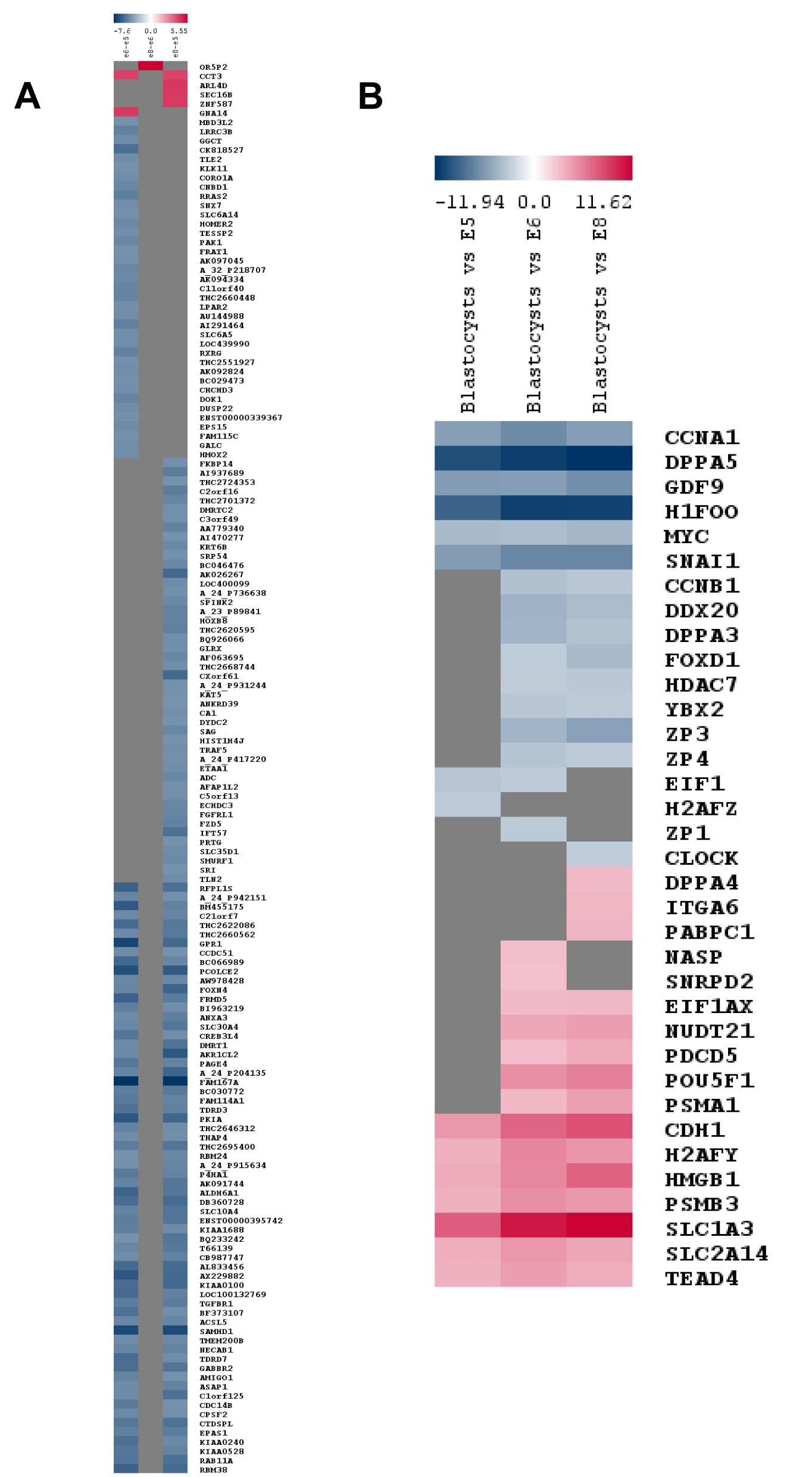

Supplement: Figure S1 — EGA signature. Heat map representation of the significant differential expression between the 5–8 single blastomeres showing novel EGA genes (A) and compared with blastocysts for previously reported genes (B). Red means an overexpression in the blastocyst samples, and blue depicts an overexpression in the single blastomere samples. The color code of the expression level is indicated at the top of the figure. (0.65 MB TIF) [file pone.0013615.s005.tif]
